# Circular test structure for the determination of piezoelectric constants of Sc_*x*_Al_1−*x*_N thin films applying Laser Doppler Vibrometry and FEM simulations^☆^

**DOI:** 10.1016/j.sna.2014.10.024

**Published:** 2015-02-01

**Authors:** P.M. Mayrhofer, H. Euchner, A. Bittner, U. Schmid

**Affiliations:** a Institute of Sensor and Actuator Systems, Vienna University of Technology, Floragasse 7, 1040 Vienna, Austria; b Institute of Materials Science and Technology, Vienna University of Technology, Karlsplatz 13, 1040 Vienna, Austria

**Keywords:** Aluminum nitride, AlN, ScAlN, Piezoelectric thin film, Vibrometry, Interferometry

## Abstract

Piezoelectric scandium aluminium nitride (Sc_*x*_Al_1−*x*_N) thin films offer a large potential for the application in micro electromechanical systems, as advantageous properties of pure AlN thin films are maintained, but combined with an increased piezoelectric actuation and sensing potential. Sc_*x*_Al_1−*x*_N thin films with *x* = 27% have been prepared by DC reactive magnetron sputtering to find optimized deposition parameters to maximize the piezoelectric constants *d*_33_ and *d*_31_. For the accurate and simultaneous measurement of these constants Laser Doppler Vibrometry has been applied and compared to finite element (FEM) simulations. The electrode design has been optimized to rotational symmetric structures enabling a 180° phase shifted excitation, so that a straight-forward comparison of experimental displacement curves with those obtained from FEM is feasible.

## Highlights

• Piezoelectric scandium aluminium nitride (Sc_*x*_Al_1−*x*_N) via sputter deposition.
• Precise determination of piezoelectric constants (*d*_33_, *d*_31_) for thin films.
• Laser Doppler Vibrometry compared to finite element simulations (FEM) using COMSOL.
• Optimized circular electrode design (Bull's eye) with two-port excitation method.
• Elastic constants of Sc_*x*_Al_1−*x*_N via density functional theory.

## 1 Introduction

Micro electromechanical systems (MEMS) based on piezoelectric thin films is an emerging research area, as such devices and systems are strongly penetrating into new market applications [1]. Aluminium nitride (AlN) is increasingly used as piezoelectric material in MEMS sensors and actuators such as accelerometers [2] or to determine the viscosity and density of liquids [3,4], surface and bulk acoustic resonators [5], atomic force microscopy (AFM) cantilevers [6], or energy harvesting systems [7]. Complementary metal-oxide-semiconductor (CMOS) compatibility, high temperature and long term stability as well as low dielectric constants are beneficial properties of AlN. For the piezoelectric constants *d*_33_ and *d*_31_ values up to 6.5 pm/V and −2.9 pm/V are reported for pure AlN thin films prepared by reactive magnetron sputtering [8–10]. A significant enhancement was achieved via incorporation of scandium (Sc) into AlN, up to 27.6 pm/V for Sc_*x*_Al_1−*x*_N thin films with *x* = 42.5% [11,12]. This increase was observed near a phase transition from a cubic to a wurtzite type crystal structure, starting at about 45%. In contrast, this work focuses on Sc_*x*_Al_1−*x*_N thin films with a fixed scandium concentration of *x* = 27%. By choosing the latter value, the formation of the cubic type crystal structure is avoided, but a strong increase of piezoelectric constants is already expected. Measurements with bulk acoustic wave (BAW) resonators based on Sc_*x*_Al_1−*x*_N with *x* = 35% showed a piezoelectric coefficient *d*_33_ = 16 pm/V, which is below the value predicted by ab initio calculations (*d*_33_ ∼ 23 pm/V) [13].

The precise knowledge of electro-mechanical constants of piezoelectric thin films is important for the design and simulation of MEMS. For example the use of finite element (FEM) software for device design requires the accurate implementation of elastic and dielectric as well as piezoelectric properties to predict precisely the performance of MEMS devices. Various measurement techniques exist for piezoelectric thin films probing either the direct piezoelectric effect, by measuring the resulting voltage upon application of mechanical stress or the inverse piezoelectric effect by measurement of the voltage induced expansion or compression. The principles of direct piezoelectric measurements were established by Lefki and Dormans for ideal cases regarding substrate clamping as well as substrate and electrode size [14]. The Berlincourt based direct measurement approach involving a reference sample with known piezoelectric properties is mentioned in this context. As this work deals with the measurement of piezoelectric constants via the indirect piezoelectric effect the discussion focuses in this direction.

Another approach, comprising the extraction of piezoelectric properties with MEMS devices, such as cantilevers, film bulk acoustic resonators (FBARs) or surface acoustic resonators (SAWs) is thoroughly covered in literature [15–18]. For an analytical evaluation of the piezoelectric properties some assumptions or important parameters need to be made or known, such as the quality factor, although the determination on device level is in principal beneficial, as it directly considers the intended application of the thin films. However, for studying new materials cantilever fabrication is a sophisticated and time consuming procedure and in general not feasible in an early stage of the work when process parameters are yet to be optimized. The evaluation of complete SAW devices or FBAR resonators via FEM simulation also requires input parameters such as the dielectric constant or sound velocity. They need to be included into the model and therefore induce further uncertainties.

As the typical intrinsic piezoelectric displacement of thin films is small (i.e. pm to nm range) measurement techniques are mostly based on optical interferometry, Laser Doppler Vibrometry (LDV) or piezoelectric force microscopy (PFM). Measurement of the piezoelectric displacement via single beam interferometry is not straight forward due to several reasons. First, the contribution of substrate bending is, compared to the change in film thickness not negligible, requesting an elaborated clamping. For a weakly clamped Si wafer covered with a piezoelectric layer Kholkin et al*.* showed that the measured piezoelectric displacement has a quadratic dependence on the electrode length. Hence, the known formula for the bending of a piezoelectric bimorph induced by the transverse piezoelectric effect is being proportional rather to *d*_31_ than to *d*_33_ [19]. Reduction of the electrode size and usage of a hard, conductive glue may be sufficient to suppress substrate bending. Moreover, a double beam interferometer for simultaneous measurement of thin film surface and substrate bottom-surface was used to minimize the impact of substrate bending [20]. However, the change in piezoelectric thin film thickness is not homogeneous below the electrode area, since the local deflection of the bottom surface of the piezoelectric thin film is also important to consider for an accurate evaluation of the displacement profile, as previously demonstrated by FEM simulations [21]. Furthermore, atomic force microscopy was used to apply the electric field via a conductive tip and measure the corresponding displacements for piezoelectric evaluation purposes [22]. However, when metallic electrodes deposited on the piezoelectric film are used the evaluation of the displacement curves is again not straight forward, but requires an in depth analysis taking the above mentioned issues into account.

The following sections discuss the deposition of highly *c*-axis oriented Sc_*x*_Al_1−*x*_N with *x* = 27% via DC reactive magnetron sputtering together with a full evaluation of both piezoelectric coefficients *d*_33_ and *d*_31_. For the measurement of piezoelectric displacement profiles via LDV new circular electrodes were designed and evaluated in comparison to COMSOL based FEM simulations. In addition, elastic properties for various concentrations *x* of Sc_*x*_Al_1−*x*_N were obtained from ab initio density functional theory (DFT) calculations.

## 2 Thin film preparation

In order to maximize the piezoelectric response of Sc_*x*_Al_1−*x*_N thin films a deposition series has been prepared via DC reactive ion sputtering. Subsequently, circular shaped platinum electrodes have been sputter-deposited and lithographically structured on top of the piezoelectric material. The thin films were deposited in a production-type sputtering system (Von Ardenne LS730S). Prior to the Sc_*x*_Al_1−*x*_N deposition, the Si(100) substrates have been cleaned by an in situ ion etching process (ISE), thus resulting in the complete removal of the native silicon surface oxide and an amorphization of the surface-near crystal structure [23]. Subsequently, thin films with a thickness of 500 nm were prepared by DC reactive magnetron sputtering from a 100 mm AlSc alloy target with a fixed ratio of 30 at.% Sc at nominally unheated substrate conditions. The base pressure prior to the deposition was kept below 4 × 10^−7^ mbar, while the other fixed process parameters are depicted in Table 1.

During the deposition series the argon ratio in the process gas (Ar/N_2_ ratio: 0%, 25%, 50%) as well as the substrate bias conditions were varied. For the latter parameter, the sputter system allows three different configurations: grounded (DC: 0 V), floating (DC: 17 V) and biased (DC: 37 V), where the depicted value for each setting is corresponding to the effective substrate self-bias. Without the exception of grounded substrate, the measured self-biase was fluctuating throughout the deposition by approximately 1 V. The deposition time was set such that the expected thin film thickness remained approximately constant at 500 nm. The scandium concentration *x* was measured by a scanning electron microscopy based energy dispersive X-ray system (EDX, Oxford Instruments X-Max 50). The determined value was *x* = (27 ± 2)% and throughout the deposition series no significant variation of *x* was observed. For the purpose of an additional calibration standard pure AlN thin films with the same equipment and sputter parameters have been deposited as described by Schneider et al., including an ion etching process prior to the deposition [23]. The circularly shaped electrode design, as illustrated in Fig. 2(a) and (c), was achieved by image reversal lithography followed by a lift-off process of platinum thin films (*t* = 100 nm). For the determination of piezoelectric constants all samples were bonded to an aluminium plate using a conductive Ag epoxy glue (Polytec EC 101).

## 3 Elastic properties of Sc_*x*_Al_1−*x*_N

**Fig. 1:**
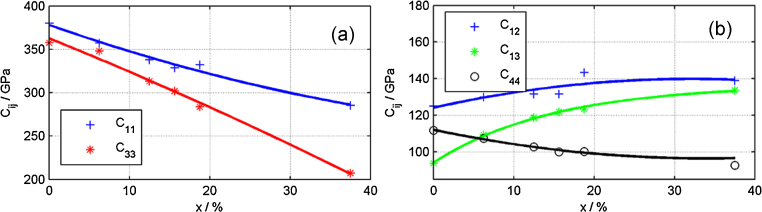
All independent elastic stiffness tensor elements *C_ij_* (in GPa) of w-Sc*_x_*Al_1−*x*_N from density functional theory.

FEM based simulations of piezoelectric structures require the complete knowledge of the anisotropic compliance tensor of Sc_*x*_Al_1−*x*_N. This work utilizes elastic constants obtained from ab initio DFT (density functional theory) calculations, as shown in Fig. 1.

The simulations were conducted using the Vienna Ab initio Simulation Package (VASP) [24], applying the projector augmented wave method and the generalized gradient approximation (PAW-GGA) [25]. To obtain representative structures, 4 × 4 × 2 supercells with altogether 128 atoms were constructed. Sc_*x*_Al_1−*x*_N was assumed to be a solid solution, in which the Sc atoms are randomly distributed on the metal sublattice. The desired Sc content was then obtained by making use of the special quasi-random structure (SQS) approach [26]. Supercells with a Sc content of 6.25%, 12.5%, 15.625%, 18.75% and 37.5% were optimized by relaxing both, lattice constant and atomic positions, using a 2 × 2 × 3 Γ-centred k-point mesh and an energy cut-off of 600 eV. The structure optimization was only stopped when residual forces and stresses were less than 0.005 eV/Å and 0.05 GPa, respectively. Next, the ground state structures were strained, using the universal independent coupling strain approach [27]. The strained configurations were again relaxed, however, with fixed lattice constants, such that only an internal relaxation of the atomic positions was conducted. From the corresponding stress–strain relationship the elastic tensor is determined by a linear least square fit procedure using single value decomposition [27]. Finally, the hexagonal projection of the elastic tensor is determined to restore symmetry [28]. This is necessary since the introduction of Sc atoms distorts the crystal matrix and therefore the supercells slightly deviate from exact hexagonal symmetry. With increasing Sc concentration in AlN the elastic constants along the main crystallite axes *C*_33_ and *C*_11_ are strongly decreasing, as shown in Fig. 1(a). Hence, a pronounced softening of the material is observed, as these two elements are related to the out of plane and in-plane elastic moduli in the material. Published experimental data on the change of *C*_33_ with *x* agree reasonably well with our data, as for instance Matloub et al. evaluated MEMS resonators via a FEM model yielding *C*_33_ = 320 GPa at *x* = 12% as well as Moreira et al., who observed a decrease down to C_33_ = 270 GPa at *x* = 15% with FBAR structures [17,29]. Furthermore, off-diagonal elements of the stiffness tensor are also changing, as depicted in Fig. 1(b). For an accurate simulation of piezoelectric based MEMS devices these changes in elastic constant need to be considered.

## 4 Measurement setup & methodology

**Fig. 2:**
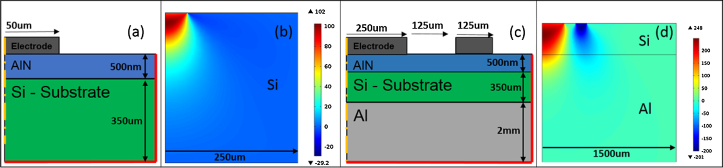
2D cross-sectional view of the rotationally symmetric electrode designs used as input for the FEM simulations (a and c). FEM results for the local displacement perpendicular to the wafer surface (b and d).

**Fig. 3:**
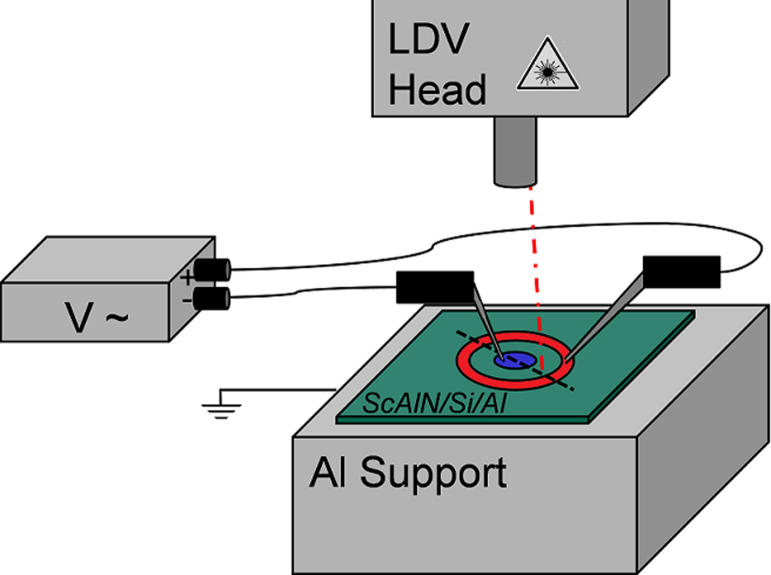
Schematic drawing of the LDV based measurement setup. Indicated is the two port deflection measurement of a Bull's eye electrode design with 180° phase shifted excitation.

Recently, Hernando et al. introduced the FEM based evaluation of interferometrically determined deflection curves from quadratic electrodes on AlN thin films. This approach takes the inherent substrate bending and the movements of bottom and top surfaces into account by a complete simulation of a structure consisting of substrate/bottom-electrode/thin film/top-electrode configuration [21]. Following their approach piezoelectric constants were determined by a measurement of the voltage induced vertical deflection via a Laser Doppler Vibrometer (Polytec MSA 400) and subsequent comparison to COMSOL based FEM simulations. All samples are conductively bonded to an aluminium plate which is fixed to the setup via a vacuum chuck and connected to electrical ground. The AC electrical excitation is applied via tungsten tips to the electrodes at frequencies of 20 kHz and 65 kHz. Fig. 2(a) and (c) show the geometries of the two most important electrode designs used within this study. The design in Fig. 2(c) consists of two electrodes: An inner ring and an outer disc-shaped element, where a 180° phase shifted voltage (amplitude: *U* = 10 V) was applied by one tungsten needle placed on each electrode. Additionally, a version of the design in Fig. 2(c) was studied which was exactly scaled by a factor of 0.5. The vertical deflection along a line scan was measured via the vibrometer, as schematically depicted in Fig. 3.

Prior to the measurements the original electrode design as introduced by Hernando et al. was optimized using the finite element software COMSOL. First, the original square shaped electrodes were changed to rotationally symmetric disc electrodes having a diameter of 100 μm resulting in a substantial reduction of computational effort thus, enabling a large accessible parameter range for the evaluation of the measurements. For each electrode geometry, the vertical piezoelectric constant was varied: *d*_33_ was increased from 1 pm/V to 18 pm/V, while the in-plane constant *d*_31_ was chosen in dependence of *d*_33_ such that *d*_31_ lies in between −*d*_33_/2 and −*d*_33_/2 + 2.2 pm/V (step sizes 0.1 pm/V). This selected range covers all meaningful *d*_31_ values, thus considering the established relationship *d*_31_ > −*d*_33_/2 for wurtzite-type crystals [30]. The FEM calculations were conducted electrostatically for a voltage of *U* = 10 V applied across the AlN layer.

Due to the rotational symmetry, the simulated structures consist of 2D boxes representing each layer as illustrated in Fig. 2. The piezoelectric thin film was meshed with at least four tetragonal elements across its thickness and with additional refinements at the electrode boundaries. This restricts the minimum film thickness, as with decreasing thickness more tetragonal elements are needed to keep an acceptable aspect ratio for the elements. Finally, the results of a complete sweep for the *z*-deflection along a centred line on the Sc_*x*_Al_1−*x*_N surface are extracted into a dataset for measurement evaluation purposes.

## 5 Results

**Fig. 4:**
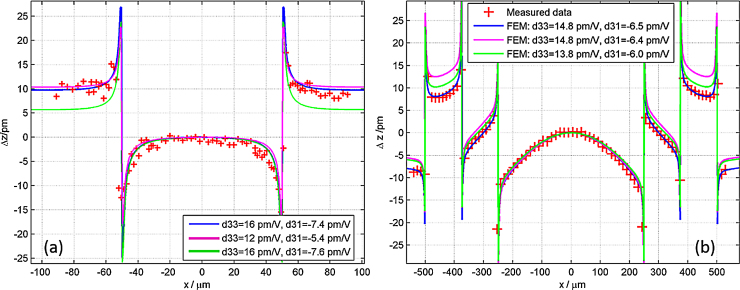
Comparison of measured displacement characteristics with those gained from FEM simulations for three different values of *d*_33_ and *d*_31_. Experimental and simulation data are vertically shifted to align at *x* = 0. Single port excitation of the simple disc in (a) and phase shifted excitation on Bull's eye in (b).

A comparison of deflection measurements and FEM curves corresponding to three pairs of piezoelectric constants is shown in Fig. 4. Qualitatively, the disc-shaped electrodes deflect similar to those having a square-shaped design where a tableau located below the top electrode is confined by an upward and downward peak at the edges. The characteristic feature for the *d*_33_ evaluation is the maximum tip-minimum tip distance, while the tableau height is depending on both the *d*_31_ and the *d*_33_ values, as previously shown [21]. As an example Fig. 4(a) shows a measurement with reasonable quality and three curves from FEM corresponding to three different values of *d*_33_ and *d*_31_. It is illustrated, that a rather large change of *d*_33_ from 16 to 12 pm/V results in nearly identical deflection (blue and magenta) curves apart from the maximum tip to minimum tip distance when keeping *d*_33_/2 + *d*_31_ constant, as this value is the critical parameter determining the tableau height. However, the accuracy in measuring the tip–tip distance is restricted to about 2 μm given by the lateral scanning resolution due to the finite laser diameter. Hence, a measurement of piezoelectric constants with small disc or square electrodes depends in principle on four points, all being affected by uncertainties resulting in a reduction of accuracy or the requirement of a large dataset.

To overcome the abovementioned problems, specifically designed Bull's eyes electrodes are introduced. The fundamental motivation is to accomplish a stronger curvature of the mentioned tableau, directly depending on *d*_33_, by an increase of the electrode area. Disadvantage of larger electrodes is the appearance of significant substrate bending together with the occurrence of eigenmodes. This is avoided via the introduction of Bull's eye type design where an outer ring is excited with the same voltage as the inner disc, but with a 180° phase shift. Thus, substrate bending can be reduced to a lower level. Therefore, FEM calculations for the Bull's eyes type electrodes include a part of the aluminium ground plate, as schematically shown in Fig. 2(c). This improves the accuracy in the determination of piezoelectric parameters, as the complete 350 μm thick silicon wafer still deflects. A FEM result for the *z*-deflection along a 2D cross section of the Bull's eyes structures is shown in Fig. 2(d) in order to illustrate the deflection close to the ground plate. In contrast, there is no deflection extending beyond the silicon wafer in the case of small simple disc electrodes, as depicted in Fig. 2(b). In these theoretical pre-investigations, the excitation voltage was set to 10 V and large piezoelectric constants were used (i.e. *d*_33_ = 15 pm/V and *d*_31_ = −7.5 pm/V) for both configurations representing a worst case scenario in mechanical deflection characteristics.

The Bull's eye type electrodes with phase shifted excitation enable a simultaneous evaluation of *d*_33_ and *d*_31_. Fig. 4(b) displays the LDV based scan along the centre of the electrode in comparison to data from FEM simulations for three *d*_33_ and *d*_31_ combinations. As illustrated, the electrode design results in significant differences between the height of inner and outer deflection plateaus even for slight variations in *d*_33_ of 1 pm/V. Thus, attributing measured data to simulations with piezoelectric constants is possible with high accuracy. Measurement and simulation data in Fig. 4(b) were shifted to the same absolute height at the electrode's centre (*x* = 0). Foremost, the plateau height itself is depending on the deviation of *d*_*31*_ from *d*_31_ = −*d*_33_/2*.* The lowest value for the piezoelectric coefficient *d*_33_, which can be determined with our Bull's eye type electrode design is limited mainly by the resolution and noise level in the Laser Doppler Vibrometer, as lower piezoelectric coefficients manifest in lower deflection amplitudes. A value for *d*_33_ of 1 pm/V results in approximately 3 pm vertical surface deflection, thus reaching the system resolution limit.

**Fig. 5:**
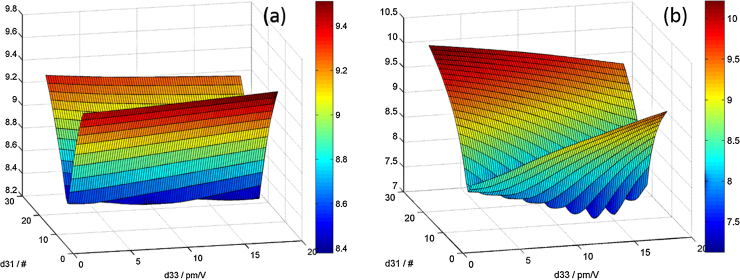
Logarithmic view of the deviation of Bull's eye based measurements from a complete set of FEM simulations; (a) is for the original design while (b) is for a Bull's eye design with half of the diameter. Simulations differ with respect to *d*_33_ and *d*_31_ where *d*_33_ is in pm/V and *d*_31_ is an index in a vector of *d*_31_ values with minimum -−*d*_33_/2 and maximum −*d*_33_/2 + 2.2 pm/V.

For a fast and precise evaluation of piezoelectric constants, a routine for automated comparison of measurements and simulations was written in MatLab. First, the absolute height of LDV data and deflection curves from FEM are aligned at a point along the scanning path, in general the centre. The best simulation is chosen on basis of a least square fitting procedure with respect to the measurement data. For a single Bull's eye electrode design Fig. 5(a) shows a graphic representation of the deviation data for each *d*_31_ and *d*_33_ combination. The discrimination along *d*_31_ is more pronounced because of the large differences in plateau height. The minimum along the *d*_33_ axis is less distinct but still present and suffices for an accurate evaluation.

Furthermore, smaller Bull's eye type electrode designs were used aiming to minimize any substrate bending effects. However, as the electrode area is reduced variations in *d*_33_ result in less pronounced changes of the curvature in the simulations. For illustration purposes, Fig. 5(b) shows the deviation values for a measurement of a specific Bull's eye type design that was scaled by factor of 0.5. A clear minimum along the *d*_33_ axis cannot be identified anymore, thus for the described evaluation procedure the smaller Bull's eye type design is not applicable and the originally introduced design, as shown in Fig. 2, performs best for the evaluation of piezoelectric constants.

**Fig. 6:**
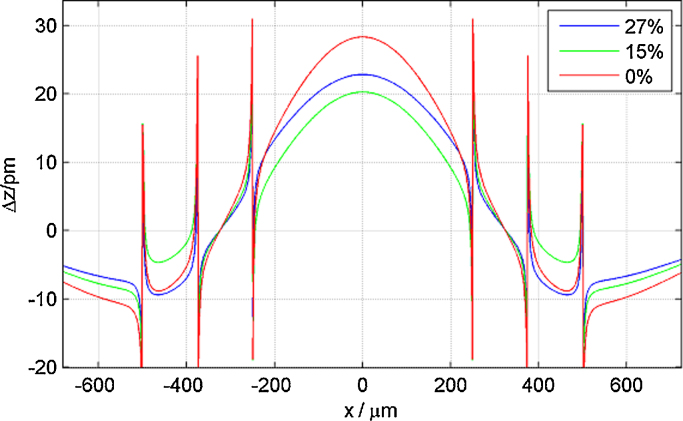
Bull's eye electrode vertical displacement for Sc*_x_*Al_1−*x*_N with elastic constants corresponding to *x* = 0%, *x* = 15% and *x* = 27% at the same piezoelectric constants.

The influence of elastic properties on the vertical displacement of Bull's eye type electrodes was studied to estimate its impact on the proposed piezoelectric constant evaluation procedure. The deflection curves for the same Bull's eye type electrode and piezoelectric constants (*d*_33_ = 15 pm/V, *d*_31_ = −7 pm/V) is shown in Fig. 6 for elastic constants corresponding to three different Scandium concentrations *x*. The depicted curves for lower *x* values exhibit a stronger deflection compared to the synthesized thin film at *x* = 27%. This behaviour is attributed to the relationship *d*_33_ ∼ e_33_/C_33_. With higher *x*, *e*_33_ increases while the decrease of *C*_33_ is more pronounced, hence leading to the strong increase in *d*_33_. An evaluation of the measurement depicted in Fig. 4(a) with elastic constants corresponding to *x* = 27% Sc_*x*_Al_1−*x*_N yields a *d*_33_ of about 14.8 pm/V which is roughly 1.6 times higher than an evaluation with elastic constants for pure AlN (*d*_33_ = 9 pm/V). This agrees reasonable well with the published ab initio based increase of *e*_33_ from 1.5 to 2.1 C/m^2^ [31].

**Fig. 7:**
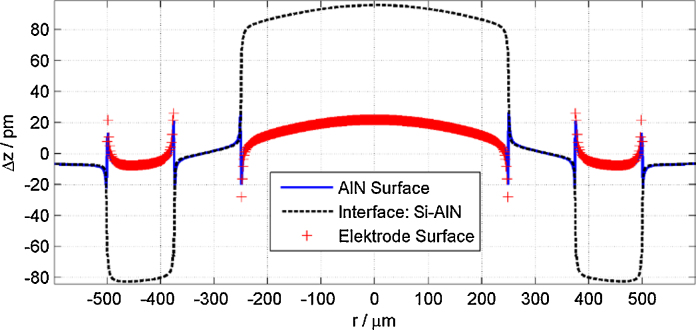
Origin of piezoelectric deflection shape from two-port Bull's Eye evaluation, pure AlN, *d*_33_ = 15 pm/V, *d*_31_ = −7.5 pm/V, *U* = 10 V – results obtained via FEM simulation.

The deflection shape of Bull's eye is further studied via evaluations of FEM simulations to explain the origin of the individual features and to illustrate the nature of the thickness change across the AlN film. Vertical displacement values at points on the Pt electrodes and between Si substrate and AlN thin film are compared with the displacement at the AlN surface, shown in Fig. 7. The polarity is such that the voltage induces a compression of the AlN in the centre disc, thus resulting in a positive deflection force at the silicon substrate and a negative deflection at the AlN surface. In contrast, the AlN layer of the outer ring shows the inverse behaviour. In addition, Fig. 7 shows, that the tableau deflection at the interface between AlN and Si is at its maximum value and the shape of the deflection is in principle such as one would expect by straight-forward considerations as a response of the film surface to the piezoelectric exaction. In contrast, the experimentally determined surface deflection of the thin film is comparatively flat. This illustrates, that the thickness change of the piezoelectric thin film is primarily located at the substrate near interface region.

It is beneficial to evaluate the deflection curves with analytical models of the piezoelectric thin film performance. An accurate analytical description may have advantages, especially when FEM simulation programs are not accessible or a large parameter range in terms of thin film composition and thickness needs to be considered. Lefki and Dormans showed that a measurement of voltage (*V*) induced deflection (Δ*L*) is not simply related to the out-of plane constant *d*_33_ via *d*_33_* = ΔL*/*V*, but rather a combination of piezoelectric and elastic material properties, as described by an effective *d*_33,*f*_:

(1.1) $$d_{33,f}=\frac{\varepsilon_{3}}{E_{3}}=d_{33}-\frac{2s_{13}}{\left(s_{11}^{E}+s_{12}^{E}\right)}d_{31}$$

With the applied electric field *E*_3_ the vertical strain *ɛ*_3_ and elastic compliance tensor elements at constant electric field $s_{ij}^{E}$. Moreover, perfect clamping parallel to the interface i.e. no strains (*ɛ*_1_ = *ɛ*_2_ = 0) and a free thin film surface, hence no stress in *z*-direction (i.e. *σ*_3_ = 0), are assumed for Eq. (1.1).

Experimental application of these requirements is further compromised by effects such as substrate bending, finite electrode size and the nature of thin film deflection. The simulation shown in Fig. 7 are based on piezoelectric constants *d*_33_ = 15 pm/V and *d*_31_ = −7.5 pm/V (elastic properties of pure AlN) yielding a *d*_33,*f*_ = 11 pm/V. Considering the applied voltage *U* = 10 V this would result in a deflection of Δ*z* = *d*_33,*f*_ · *U* = 110 pm, which strongly overestimates the surface deflection. The reason for this overestimation is on the one hand the implied condition of a perfectly clamped substrate and on the other hand the relatively small electrode size, inducing clamping throughout the whole electrode area. These results have also implication for other indirect-piezoelectric measurement techniques, such as piezoelectric deflection measurements via piezoresponse atomic force microscopy (AFM/PFM), as it illustrates that piezoelectric constants simple cannot be calculated from the deflection amplitude of electrodes.

## 6 Discussion

**Fig. 8:**
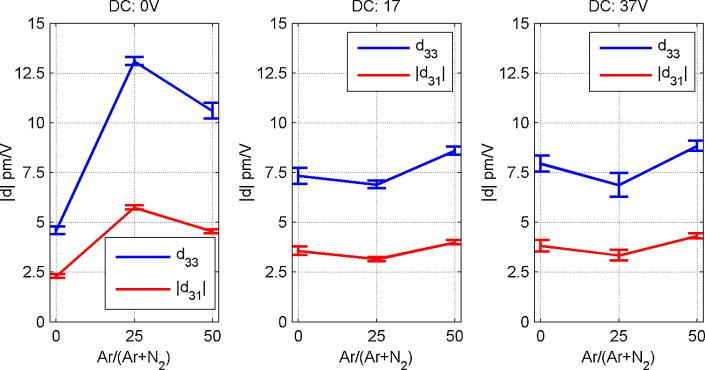
Piezoelectric constants of Sc*_x_*Al_1−*x*_N with *x* = 27% with varying deposition parameters: substrate bias, depicted in the upper right of each graph and ratio of argon in the process gas on the axis.

To verify the applicability of the novel test structure, pure AlN thin films with well investigated material properties have been measured first by employing the presented methodology. The resulting piezoelectric coefficients for these AlN samples were *d*_33_ = 4.2 pm/V and *d*_31_ = −1.9 pm/V, thus agreeing excellent with those reported by Hernando et al*.* [21]. Next, the piezoelectric constants of Sc_*x*_Al_1−*x*_N thin films (*x* = 27%) with varying deposition parameters as described above have been evaluated with the Bull's eye type electrode design. Fig. 8 shows the resulting values for *d*_33_ and *d*_31_ with varying Ar ratio in the process gas and varying substrate bias conditions. Each depicted data point is the average of about four measurements taken at excitation frequencies of 25 kHz and 65 kHz. Error bars are based on the corresponding standard deviation, but minimum values of 0.25 pm/V for *d*_33_ and 0.1 pm/V for *d*_31_ were estimated originating from the uncertainty when comparing measurements and FEM simulations.

A maximum in both piezoelectric constants is obtained for 25% Ar and grounded substrate configuration. Therefore, these deposition conditions can be regarded as the optimum to achieve highest actuation potential of Sc_*x*_Al_1−*x*_N thin films within the investigated parameter range. They yield *d*_33_ = 13.2 pm/V and *d*_31_ = −5.8 pm/V. These results agree well with earlier measurements of *d*_33_ of Sc_*x*_Al_1−*x*_N (*d*_33_ ∼ 15 pm/V) [11]. In addition, the values for *d*_33_ and *d*_31_ are in line with results from ab initio calculations that predict values for *x* = 27% of about *d*_31_ ∼ 7 pm/V and *d*_33_ ∼ 17 pm/V [13].

Optimized parameters for the DC reactive sputter deposition of AlN thin films are crucial in order to obtain highly *c*-axis oriented thin films with a maximized piezoelectric response [22]. For Sc_*x*_Al_1−*x*_N thin films with a low concentration of *x* = 6% the sputtering power showed to have an impact on the degree of *c*-axis orientation and thus, on the piezoelectric response of the films [32]. On the other hand, for Sc_*x*_Al_1−*x*_N thin films with *x* up to 15% we previously showed that the Ar ratio in the process gas has a particular influence on crystalline quality and *c*-axis orientation [33]. Ar addition leads to higher ad-atom mobility during the sputter process which in turn results in a lower amount of defects in crystallites and a highly pronounced *c*-axis oriented growth. Hence, the requirement of Ar admixture for maximized piezoelectric response is in-line with these results. Additionally, the substrate bias also proved to have an influence, as for pure AlN thin film deposition an enhancement of the *c*-axis orientation was shown for decreasingly negative substrate self-bias [34]. The observed behaviour for the deposition of Sc_*x*_Al_1−*x*_N is similar though in this work, the substrate bias could not be further decreased due to system limitations.

## 7 Conclusion

This work studies the piezoelectric behaviour of DC sputter deposited Sc_*x*_Al_1−*x*_N (*x* = 27%) thin films. Ar ratio in the process gas and substrate bias were varied and found to have a significant impact on the piezoelectric constants *d*_33_ and *d*_31_. For the advanced determination of piezoelectric constants test structures are reported requiring low fabrication effort, with optimized platinum electrode designs for FEM evaluation of LDV based vertical deflection measurements. The resulting Bull's Eye shaped electrodes with rotational symmetry enable a precise and simultaneous straight-forward evaluation of *d*_33_ and *d*_31_ by comparing experimental and theoretical results. For FEM based calculation of piezoelectric deflection curves elastic constants from ab initio calculation have been used. The piezoelectric response (*d*_33_ = 13.2 pm/V and *d*_31_ = −5.8 pm/V) was maximized for an argon concentration of 25% and for grounded substrate conditions. Thus, a further step towards Sc_*x*_Al_1−*x*_N based piezoelectric MEMS devices with enhanced electromechanical properties is provided.

## Figures and Tables

**Table 1:** Process parameters for the ISE process and the Sc_*x*_Al_1−*x*_N thin film synthetization (process pressure in the deposition chamber *p*, plasma power density *P*, process time *t*, argon gas flow *v*_Ar_ and electrode distance *d*).

ISE					Deposition			
*p*/Pa	*P*/W cm^−2^	*t*/s	*d*/mm	*v*_Ar_/sccm	*p*/Pa	*P*/W cm^−2^	*t*/s	*d*/mm
0.6	6.4	60	65	60	0.25	5.1	800–1300	65
